# Multimorbidity and complex multimorbidity in Brazilians with severe obesity

**DOI:** 10.1038/s41598-023-43545-5

**Published:** 2023-10-03

**Authors:** Ana Paula dos Santos Rodrigues, Sandro Rogério Rodrigues Batista, Annelisa Silva e Alves Santos, Andrea Batista de Sousa Canheta, Bruno Pereira Nunes, Andréa Toledo de Oliveira Rezende, Cesar de Oliveira, Erika Aparecida Silveira

**Affiliations:** 1 Superintendence of Health Care, Goiás State Health Department, Goiânia, Goiás Brazil; 2 Department of Internal Medicine, School of Medicine, Federal University of Goiás, Goiânia, Goiás Brazil; 3 Primary Healthcare Office, Federal District State Health Department, Brasília, Distrito Federal Brazil; 4 Graduate Program in Health Sciences, Federal University of Goiás, Goiânia, Goiás Brazil; 5 Faculty of Education, Federal University of Goiás, Goiânia, Goiás Brazil; 6 Graduate Program in Nursing, Federal University of Pelotas, Pelotas, Brazil; 7 Department of Epidemiology & Public Health, University College London, London, UK

**Keywords:** Endocrinology, Medical research

## Abstract

To investigate the prevalence of multimorbidity and complex multimorbidity and their association with sociodemographic and health variables in individuals with severe obesity. This is a baseline data analysis of 150 individuals with severe obesity (body mass index ≥ 35.0 kg/m^2^) aged 18–65 years. The outcomes were multimorbidity and complex multimorbidity. Sociodemographic, lifestyle, anthropometric and self-perceived health data were collected. Poisson multiple regression was conducted to identify multimorbidity risk factors. The frequency of two or more morbidities was 90.7%, three or more morbidities was 76.7%, and complex multimorbidity was 72.0%. Living with four or more household residents was associated with ≥ 3 morbidities and complex multimorbidity. Fair and very poor self-perceived health was associated with ≥ 2 morbidities, ≥ 3 morbidities and complex multimorbidity. A higher BMI range (45.0–65.0 kg/m^2^) was associated with ≥ 2 morbidities and ≥ 3 morbidities. Anxiety (82.7%), varicose veins of lower limbs (58.7%), hypertension (56.0%) were the most frequent morbidities, as well as the pairs and triads including them. The prevalence of multimorbidity and complex multimorbidity in individuals with severe obesity was higher and the risk for multimorbidity and complex multimorbidity increased in individuals living in households of four or more residents, with fair or poor/very poor self-perceived health and with a higher BMI.

## Introduction

Severe obesity (class II and III obesity) is a public health problem. Its prevalence has been increasing in an alarming rate worldwide. A study carried out in the United States projected that nearly 1 in 4 adults will have severe obesity by 2030 (24.2%; 95% CI 22.9 to 25.5) and its prevalence will reach more than 25%^1^. Another study in Spain found that 3.9% of adults suffered from class II obesity and over, affecting 1.32 million people^2^. In Brazil, 8.9 million adults had severe obesity in 2014 and its prevalence in women was 5.3% and in men 3.8%^3^.

In addition to the negative health impacts in individuals, severe obesity is often associated with several other morbidities^4^, especially a cardiometabolic cluster including hypertension, dyslipidemia, and diabetes^5,6^. In individuals with severe obesity, besides the elevated occurrence of cardiometabolic morbidities, osteoarticular and mental health problems are highly prevalent^5^. Among these, the most common are depression^7^ and anxiety^8^, highlighting that severe obesity is accompanied by a complex health scenario^5^.

In this context, the study of multimorbidity, that is, the presence of two or more morbidities in the same individual^9,10^, is important for this population, especially regarding the assessment of which morbidities occur more frequently in individuals with obesity^5,11,12^. This information is essential to the potential clinical interrelationship among morbidities and, consequently, to the associated pharmacological and non-pharmacological treatment approaches in this context^13^.

The concept of complex multimorbidity has been used to define the co-occurrence of three or more chronic conditions that affect three or more different body systems or different domains. This construct leads to lower prevalence compared to the traditional definition of multimorbidity, and is argued that it may also identify higher-need patients that demand care from different health providers^14^. Coordinating health care among the various medical specialists is a critical issue, thus, assessing complex multimorbidity may enable disease management and health policy planning^15^. People with severe obesity (BMI ≥ 35 kg/m^2^) are at greater risk for several morbidities and that they may account for a high proportion of the healthcare workload. However, few investigations contemplate this population, making it difficult to obtain a more robust picture on this subject^5,16^. Thus, it is important to investigate complex multimorbidity in this population.

Previous studies^17,18^ have identified that some sociodemographic factors and lifestyle are associated with multimorbidity and complex multimorbidity such as age^19^, sedentary lifestyle^20^, socio-economic inequalities^21^, daily moderate intake of alcohol^22^. However, there are few studies that identified the sociodemographic characteristics involved, the prevalence, and the pattern of morbidities in individuals with severe obesity. Understanding these factors is important to provide an integrated care approach, mainly because these morbidities are interrelated in a complex way and need to be managed in a coordinated and timely manner^23^. Additionally, there are still few studies that assess beyond the co-occurrence of morbidities. Therefore, this study aimed to investigate the prevalence of multimorbidity and complex multimorbidity and their association with sociodemographic, lifestyle and health variables in individuals with severe obesity. We also aimed to identify the most frequent morbidities, pairs, and triads according to BMI class.

## Results

We analyzed data from 150 individuals with severe obesity with mean BMI 46.1 (± 6.5) kg/m^2^ and mean number of morbidities of 4.4 (± 2.4). The frequency of two or more morbidities was 90.7% (95%CI 84.8–94.8) (n = 136), three or more morbidities was 76.7% (95%CI 69.1–83.2) (n = 115), and complex multimorbidity was 72.0% (95%CI 64.7–79.2) (n = 108).

The mean age was 39.6 (± 8.8) years while the predominant age group was 35–44 years (44.0%), 85.3% were women, and 55.3% had brown skin color. Considering education and professional status, 67.3% had 9 or more years of schooling, 51.1% had lower per capita family income, and 68.7% were working in formal/informal/self-employed jobs. In addition, 52.0% lived in households with 4 or more residents, 67.3% reported never smoking, 70.2% had no binge drinking episodes in the last year, and 41.3% reported fair self-perceived health. Age group was associated with ≥ 3 morbidities, while household residents’ number, health self-perception, and BMI range were associated with ≥ 2 and ≥ 3 morbidities and also with complex multimorbidity (Table 1).

**Table 1:** Multimorbidity occurrence according to sociodemographic characteristics, lifestyle and self-perceived health in individuals with severe obesity.

Variables	N (%)	≥ 2 morbidities		≥ 3 morbidities		Complex multimorbidity	
		N (%)	PR (95%CI)	N (%)	PR (95%CI)	N (%)	PR (95%CI)
Gender			p = 0.694***		p = 1.000***		p = 0.666*
Female	128 (85.3)	115 (89.8)	1	98 (76.6)	1	93 (72.7)	1
Male	22 (14.7)	21 (95.4)	1.06 (0.95–1.18)	17 (77.3)	1.01 (0.79–1.29)	15 (68.2)	1.07 (0.78–1.45)
Age group (in years)			p = 0.099***		p = 0.010**		p = 0.245*
18–34	44 (29.3)	37 (84.1)	1	29 (65.9)	1	28 (63.6)	1
35–44	66 (44.0)	60 (90.9)	1.08 (0.93–1.26)	50 (75.8)	1.15 (0.89–1.48)	48 (72.7)	1.14 (0.87–1.50)
45–65	40 (26.7)	39 (97.5)	1.16 (1.01–1.33)	36 (90.0)	1.37 (1.08–1.73)	32 (80.0)	1.26 (0.96–1.65)
Schooling (in years)			p = 0.550***		p = 0.068*		p = 0.505
0–8	49 (32.7)	46 (93.9)	1.05 (0.95–1.16)	42 (85.7)	1.19 (1.00–1.40)	37 (75.5)	1.07 (0.88–1.32)
≥ 9	101 (67.3)	90 (89.1)	1	73 (72.3)	1	71 (70.3)	1
Per capita family income (R$)			p = 0.583***		p = 0.657*		p = 0.566*
Lower income (0–500.00)	76 (51.1)	70 (92.1)	1.03 (0.93–1.15)	57 (75.0)	1	53 (69.7)	1
Higher income (500.01–5000.00)	73 (48.9)	65 (89.0)	1	57 (78.1)	1.04 (0.87–1.24)	54 (74.0)	1.06 (0.87–1.30)
Marital status			p = 0.914***		p = 0.524*		p = 0.654***
Single	39 (26.0)	36 (92.3)	1.03 (0.92–1.16)	32 (82.0)	1.11 (0.92–1.35)	29 (74.4)	1.07 (0.85–1.34)
Married	95 (63.3)	85 (89.5)	1	70 (73.7)	1	66 (69.5)	1
Widowed/ divorced	16 (10.7)	15 (93.8)	1.05 (0.91–1.21)	13 (81.2)	1.10 (0.84–1.44)	13 (81.2)	1.16 (0.89–1.53)
Skin color			p = 0.673***		p = 0.963***		p = 0.823*
White	46 (30.7)	42 (91.3)	1.06 (0.87–1.30)	36 (78.3)	1	33 (71.7)	1.08 (0.76–1.53)
Brown	83 (55.3)	76 (91.6)	1.07 (0.89–1.29)	63 (75.9)	1.03 (0.85–1.25)	61 (73.5)	1.10 (0.79–1.53)
Black	21 (14.0)	18 (85.7)	1	16 (76.2)	1.00 (0.77–1.31)	14 (66.7)	1
Occupation			p = 0.227***		p = 0.217*		p = 0.215*
Formal/informal/self-employed	103 (68.7)	91 (88.4)	1	76 (73.8)	1	71 (68.9)	1
Unemployed/retired/pensioner	47 (31.3)	45 (95.7)	1.08 (0.99–1.19)	39 (83.0)	1.12 (0.94–1.34)	37 (78.7)	1.14 (0.94–1.39)
Household resident number			p = 0.049***		p = 0.003*		p = 0.001*
< 4	72 (48.0)	67 (85.9)	1	52 (66.7)	1	47 (60.3)	1
≥ 4	78 (52.0)	69 (95.8)	1.12 (1.01–1.24)	63 (87.5)	1.31 (1.10–1.57)	61 (84.7)	1.41 (1.14–1.73)
Smoking			p = 0.387***		p = 0.317*		p = 0.292*
Never smoked	101 (67.3)	93 (92.1)	1.05 (0.93–1.18)	75 (74.3)	1	70 (69.3)	1
Smoker/former smoker	49 (32.6)	43 (87.8)	1	40 (81.6)	1.10 (0.92–1.31)	38 (77.6)	1.12 (0.92–1.37)
Binge drinking			p = 0.062***		p = 0.180***		p = 0.292***
None	99 (70.2)	91 (91.9)	1.23 (0.94–1.59)	78 (78.8)	1.31 (0.90–1.91)	71 (71.7)	1.20 (0.82–1.75)
1–3 times per month	22 (15.6)	21 (95.4)	1.27 (0.97–1.67)	18 (81.8)	1.36 (0.90–2.05)	18 (81.8)	1.36 (0.90–2.05)
> 3 times per month	20 (14.1)	15 (75.0)	1	12 (60.0)	1	12 (60.0)	1
Health self-perception			p < 0.001***		p < 0 0.001**		p < 0.001***
Very good/good	42 (28.0)	31 (73.8)	1	23 (54.8)	1	22 (52.4)	1
Fair	62 (41.3)	60 (96.8)	1.31 (1.09–1.58)	50 (80.6)	1.47 (1.09–1.99)	44 (71.0)	1.36 (0.97–1.88)
Poor/very poor	46 (30.7)	45 (97.8)	1.32 (1.10–1.60)	42 (91.3)	1.67 (1.25–2.23)	42 (91.3)	1.74 (1.29–2.36)
BMI range			p = 0.009***		p = 0.005*		p = 0.015*
35.0–44.9 kg/m^2^	76 (50.7)	64 (84.2)	1	51 (67.1)	1	48 (63.2)	1
45.0–65.0 kg/m^2^	74 (49.3)	72 (97.3)	1.16 (1.04–1.28)	64 (86.5)	1.29 (1.07–1.55)	60 (81.1)	1.28 (1.05–1.58)

95%CI: 95% confidence interval; BMI: body mass index; PR: prevalence ratio.

*Chi-squared, **Tendency chi-squared, ***Fisher’s exact test.

Significant values are in bold.

After adjustment in multiple regression analysis, living with four or more household residents was associated with ≥ 3 morbidities (PR: 1.31, 95%CI 1.09–1.56), and complex multimorbidity (PR: 1.39, 95%CI 1.11–1.74). Fair self-perceived health was associated with ≥ 2 morbidities (PR: 1.28, 95%CI 1.07–1.52), ≥ 3 morbidities (PR: 1.45, 95%CI 1.09–1.92), and complex multimorbidity (PR: 1.54, 95%CI 1.04–2.29). The same was observed for poor/very poor self-perceived health (≥ 2 morbidities: PR: 1.29, 95%CI 1.08–1.54; ≥ 3 morbidities: PR: 1.60, 95%CI 1.21–2.12, complex multimorbidity (PR: 2.08, 95%CI 1.44–3.01). A greater BMI range (45.0–65.0 kg/m^2^) was associated to ≥ 2 morbidities (PR: 1.16, 95%CI 1.04–1.29) and ≥ 3 morbidities (PR: 1.29, 95%CI 1.08–1.54) (Table 2).

**Table 2:** Multiple regression analysis of multimorbidity and associated factors in individuals with severe obesity.

Variables	≥ 2 morbidities		≥ 3 morbidities		Complex multimorbidity	
	Adjusted PR (95%CI)	p value*	Adjusted PR (95%CI)	p value*	Adjusted PR (95%CI)	p value*
Age group (in years)						
18–34	1		1		–	–
35–44	1.07 (0.94–1.24)	0.263	1.12 (0.89–1.40)	0.340	–	–
45–65	1.05 (0.93–1.20)	0.086	1.08 (0.86–1.36)	0.502	–	–
Schooling (in years)						
0–8	–	–	1.10 (0.92–1.30)	0.301	–	–
≥ 9	–	–	1		–	–
Household resident number						
< 4	1		1		1	
≥ 4	1.10 (1.00–1.22)	0.057	1.31 (1.09–1.56)	**0.003**	1.39 (1.11–1.74)	**0.004**
Binge drinking						
None	1.18 (0.95–1.48)	0.135	1.20 (0.88–1.63)	0.255	–	–
1–3 times per month	1.25 (0.98–1.58)	0.069	1.28 (0.90–1.82)	0.173	–	–
> 3 times per month	1		1		–	–
Self-perceived health						
Very good/good	1		1		1	
Fair	1.28 (1.07–1.52)	**0.006**	1.45 (1.09–1.92)	**0.010**	1.54 (1.04–2.29)	**0.032**
Poor/very poor	1.29 (1.08–1.54)	**0.004**	1.60 (1.21–2.12)	**0.001**	2.08 (1.44–3.01)	** < 0.001**
BMI range						
35.0–44.9 kg/m^2^	1		1		1	
45.0–65.0 kg/m^2^	1.16 (1.04–1.29)	**0.006**	1.29 (1.08–1.54)	**0.004**	1.18 (0.95–1.47)	0.135

PR: prevalence ratio.

Significant values are in bold.

*Wald statistics.

Anxiety (82.7%), varicose veins of lower limbs (58.7%) and hypertension (56.0%) were the most frequent morbidities, followed by dyslipidemia (44.8%) and depression (31.3%). The combination of anxiety-hypertension (51.3%), anxiety-varicose veins of lower limbs (50.0%), and anxiety-dyslipidemia (36.7%) were the most common pairs of morbidities. Among triads of morbidities, anxiety-varicose veins of lower limbs-hypertension (31.3%), anxiety-hypertension-dyslipidemia (28.7%), and anxiety-varicose veins of lower limbs-dyslipidemia (22.0%) had the highest frequencies (Table 3).

**Table 3:** Morbidity frequency and top 10 morbidity pairs and triads in individuals with severe obesity.

Morbidity	N (%)
Anxiety	124 (82.7)
Varicose veins of lower limbs	88 (58.7)
Hypertension	84 (56.0)
Dyslipidemia	65 (44.8)
Depression	47 (31.3)
Arthritis/arthrosis	32 (21.5)
Urinary incontinence	30 (20.0)
Sleep apnea	27 (18.0)
Diabetes mellitus	27 (18.0)
Gastroesophageal reflux	25 (16.7)
Liver disease^a^	23 (15.3)
Respiratory disease^b^	22 (14.7)
Thyroid disease^c^	22 (14.7)
Infertility	19 (12.9)
Cardiovascular disease	15 (10.0)
Stroke	4 (2.7)
Polycystic ovary syndrome	4 (2.7)
Osteoporosis	3 (2.0)
Cancer	2 (1.3)
Pairs	
Anxiety + hypertension	77 (51.3)
Anxiety + varicose veins of lower limbs	75 (50.0)
Anxiety + dyslipidemia	55 (36.7)
Hypertension + dyslipidemia	48 (32.0)
Anxiety + depression	45 (30.0)
Varicose veins of lower limbs + dyslipidemia	37 (24.7)
Anxiety + arthritis/arthrosis	31 (20.7)
Anxiety + urinary incontinence	28 (18.7)
Hypertension + depression	28 (18.7)
Anxiety + diabetes mellitus	26 (17.3)
Triads	
Anxiety + varicose veins of lower limbs + hypertension	47 (31.3)
Anxiety + hypertension + dyslipidemia	43 (28.7)
Anxiety + varicose veins of lower limbs + dyslipidemia	33 (22.0)
Anxiety + hypertension + depression	28 (18.7)
Varicose veins of lower limbs + hypertension + dyslipidemia	28 (18.7)
Anxiety + varicose veins of lower limbs + depression	24 (16.0)
Anxiety + dyslipidemia + depression	23 (15.3)
Anxiety + hypertension + arthritis/arthrosis	22 (14.7)
Anxiety + varicose veins of lower limbs + arthritis/arthrosis	20 (13.0)
Varicose veins of lower limbs + hypertension + depression	16 (10.7)

^a^Fatty liver disease, cirrhosis.

^b^Asthma, bronchitis.

^c^Hypothyroidism, hyperthyroidism.

**Figure 1:**
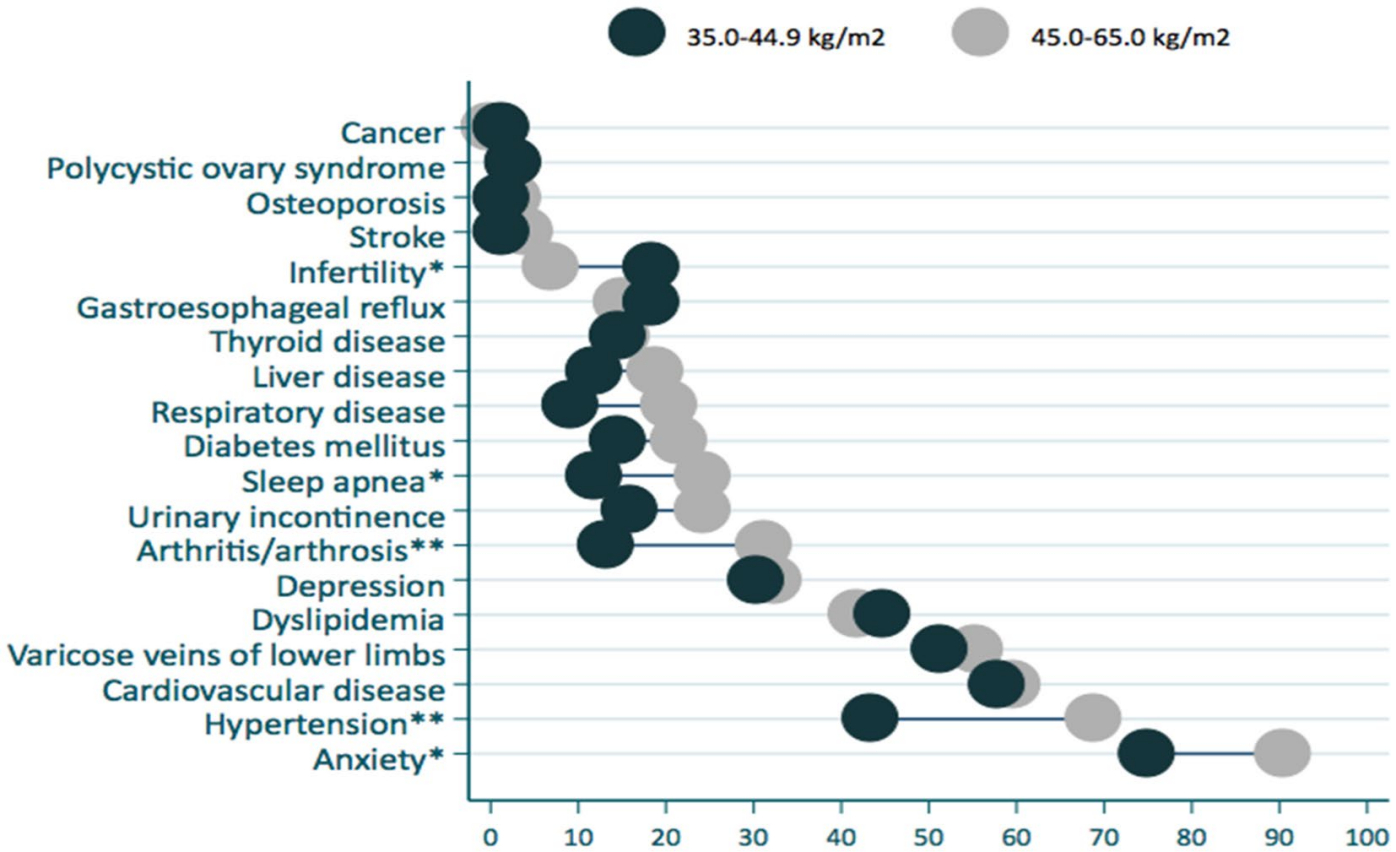
Prevalence of morbidities stratified by body mass index ranges of individuals with severe obesity. *p < 0.05, **p < 0.01.

Individuals in the BMI range of 45.0 to 65.0 kg/m^2^ had significantly increased frequency of some morbidities compared to individuals with lower BMI (35.0 to 44.9 kg/m^2^), such as sleep apnea (24.3% vs. 11.8%, p < 0.05), arthritis/arthrosis (31.1% vs. 13.2%, p < 0.01), hypertension (68.9% vs. 43.4%, p < 0.01) and anxiety (90.5% vs. 75%, p < 0.05). For infertility the opposite was observed (26.3% vs. 73.7%, p < 0.05) (Fig. 1).

## Discussion

Few studies have investigated complex multimorbidity in individuals with severe obesity. Our study provides valuable evidence for middle-income countries as few studies have investigated exclusively severe obesity and the co-occurrence of several morbidities and its associated factors in such context. Our results have shown that the prevalence of multimorbidity and complex multimorbidity in individuals with severe obesity are extremely high and the risk for multimorbidity has increased in individuals living in households of four or more residents, with fair or poor/very poor self-perceived health and with greater BMI. Additionally, we observed that cardiometabolic and mental health disorders were among the most frequent morbidities, such as hypertension, dyslipidemia, anxiety, and depression, as well as the combination of them. This scenario reveals the complex health status of individuals with severe obesity and the importance of developing clinical approaches capable of addressing not only the metabolic disorders, but also the mental health issues associated to severe obesity.

In the present study, we observed a high prevalence of multimorbidity (90.7% for two or more conditions and 76.7% for three or more conditions), as reported in other studies with individuals with severe obesity^5,16^. A Canadian study with 500 severely obese adults found 95.4% and 85.8% multimorbidity considering two and three chronic conditions, respectively^5^. Another study carried out in the United States, considering a list of 82 diseases, found a prevalence of 3 or more morbidities around 70% for obesity classes II and III^24^, which was similar to the present study. In the United Kingdom, results from a cohort demonstrated an increasing prevalence of multimorbidity as the BMI range rises, with emphasis on three times greater risk of ≥ 3 chronic conditions in individuals with BMI ≥ 40 kg/m^2^ compared to eutrophic individuals^16^. Divergences in the prevalence of multimorbidity are likely to be related to the use of different definitions of the outcome, the types and numbers of chronic conditions included in this assessment. Furthermore, the observation of greater risk of multimorbidity in higher degrees of obesity has been confirmed in several studies^11,16,24–26^. The inclusion of obesity in multimorbidity indices, sometimes left out, has been debated as a relevant point for epidemiological surveillance^11^. In this context, the importance of evaluating this outcome at higher levels of BMI is highlighted, given the exponential increase in severe obesity in recent years.

Age has been consistently associated with multimorbidity^27^, as the studies frequently report increasing prevalence with age^27,28^, and higher numbers in older persons compared (55–98%) to the whole population (20–30%)^27^. In our study, the prevalence of multimorbidity was higher in individuals aged 45–65 years (90%) when compared to 18–34 years (65.9%) only for ≥ 3 morbidities. We highlight the impact of severe obesity on the prevalence of multimorbidity demonstrated by the elevated estimates in the young adults with severe obesity in our study.

In this study, individuals with severe obesity living with four or more household residents had a higher risk of having three or more morbidities and complex multimorbidity. Data on family structure have shown that not living with children is associated with greater risk of multimorbidity^29,30^, however, investigations regarding the size of the family were not found. Family involvement has an important role in multimorbidity patient care, especially for the older adults. For younger individuals there is a lack of information regarding family structure and support in patient self-management of chronic diseases^13,29^. However, some negative experiences regarding the family involvement have been reported. Barriers to self-care (e.g. a family member refusal to eat the type of food the patient would like to eat) and responsibilities to multiple family members (multiple caregiver role) imposing competing demands on the patient’s time may result in poor self-management, nevertheless this topic is not clearly understood and needs further investigation^31,32^.

Concerning the health self-perception, in this study, the poor/very poor self-perceived health was associated with ≥ 2 morbidities, ≥ 3 morbidities, and complex multimorbidity. Several studies have demonstrated association of multimorbidity with fair and poor self-rated health in different populations, such as middle-aged and older individuals (OR = 3.4, 95% IC = 2.3–5.1)^33^, adults (PR = 3.70, 95% CI 2.73 to 5.00)^34^, older women^35^, Brazilian rural workers (OR 2.10, 95% CI 1.52–2.91)^36^, and persons aged 50 and older from 16 European nations (Adjusted OR = 2.13, 95% CI = 2.03–2.24)^37^. For complex multimorbidity, the same association was observed in Brazilian rural workers (OR 2.25, 95% CI 1.49–3.38)^36^.

Cardiometabolic morbidities are common in individuals with obesity^6^, resulting from excess body fat in different body regions, including the visceral region^38^. Varicose veins of lower limbs, hypertension and dyslipidemia were among the most frequent morbidities found in our sample, as expected. Concurrent cardiometabolic morbidities in this population collaborate to a higher risk of cardiovascular events, one of the major causes of death globally. The early identification of these outcomes contributes to the reduction of cardiovascular morbidity and mortality in individuals with obesity, especially severely obese ones^6,39,40^.

Another set of morbidities with common occurrence in obesity refers to mental health disorders, such as anxiety and depression^41^. Our results emphasize a high prevalence of anxiety and depression as they are among the most common morbidities, both alone and in pairs and triads. The high prevalence of mental health disorders in obesity could be attributed to both as a result of the disease itself, which carries a negative stigma for the individual, and as the multimorbidity associated with obesity^42^. Furthermore, individuals with multimorbidity may be at greater risk of developing or worsening mental health^43,44^. If not properly managed, mental health disorders may significantly interfere in the patient's compliance with obesity and physical multimorbidity treatments, further worsening their health status^45^.

The present study has several strengths, such as its methodological quality and control during data collection; a wider list of morbidities/chronic conditions than previous studies that also used self-reported conditions; and the investigation of an important public health problem, such as severe obesity, that has been poorly investigated. As a potential limitation, we could mention the age range that did not include elderly individuals (> 65 years), and the use of self-reported data based on previous medical diagnosis. Despite many studies using this methodology to assess multimorbidity, and some self-reported information have been validated^46,47^, it may still present some bias, especially for mental health disorders^48^.

Our study has provided important data that could help improve prevention of diseases and healthcare of individuals with severe obesity. Multimorbidity, as a condition that affects populations across the world and, especially, people with obesity and severe obesity, has attracted the attention of researchers and clinicians. In this context, future studies should investigate multimorbidity applying weighted indexes that have been recently explored in some research. These indexes may provide a better understanding of the impact of multimorbidity on the functional status of individuals and enable tailored clinical decision-making, and guide interventions to maximize and preserve physical functioning.

## Conclusion

Individuals with severe obesity had a high prevalence of multimorbidity and complex morbidity. Multimorbidity and complex multimorbidity were associated with living in households of four or more residents and fair or poor/very poor self-perceived health, while BMI of 45–65 kg/m^2^ was associated only with multimorbidity. The most common morbidities were cardiometabolic and mental health disorders, as well as the pairs and triads including them.

## Methods

We analyzed baseline data of individuals with severe obesity from a randomized clinical trial entitled “Effect of Nutritional Intervention and Olive Oil in Severe Obesity: Randomized Controlled Trial-DieTBra Trial” (registered at ClinicalTrials.gov, NCT02463435). The research was conducted at the Clinical Research Unit (CRU) from Clinics Hospital, Federal University of Goiás, Goiânia, Goiás State, Brazil. We recruited individuals with BMI ≥ 35 kg/m^2^, aged 18 to 65 years old, and living in Goiânia and the metropolitan area. Exclusion criteria were to have already performed bariatric surgery, pregnancy, actual nutritional treatment for weight loss or in the previous 2 years, anti-obesity or anti-inflammatory drugs use, HIV/AIDS, heart/kidney/hepatic insufficiency, chronic obstructive pulmonary disease, and cancer. More detailed information on the DieTBra Trial can be found in previous publications^6,49,50^.

This study was approved by the Ethics Committee on Research with Humans of the Clinical Hospital/Federal University of Goiás under protocol number 747.792 and was conducted in accordance with the principles outlined in the Declaration of Helsinki. Informed consent was obtained from all participants included in the study, and the patient anonymity was preserved.

Structured questionnaires were tested and standardized in a pilot study for data collection. At baseline, we collected the following sociodemographic and health data: sex, age, schooling, per capita family income, marital status, skin color, work status, household number of residents, weight, height, BMI, smoking, alcohol consumption, health self-perception, and morbidities.

Participants’ body weight was measured to the nearest 0.05 kg using a calibrated digital scale (Welmy^®^) with capacity of 200 kg. Height was measured to the nearest 0.1 cm using a stadiometer. BMI (kg/m^2^) was calculated dividing the body mass (kg) by the squared height (m^2^). Severe obesity was defined as BMI ≥ 35 kg/m^2^ (NCD Risk Factor Collaboration, 2016).

Smoking habit was assessed by asking if the participant smoked or had ever smoked (cigarette, cigar, or pipe). Participants were classified as never smokers, former smokers, or current smokers. Alcohol consumption was assessed as binge drinking episodes (consumption of 5 or more drinks on at least 1 occasion) in the last year through an adapted version of the questionnaire of the Gender, Alcohol and Culture: An International Study (GENACIS) project^51^. Self-perceived health was assessed by asking: “In general, how do you rate your health: very good, good, fair, poor, or very poor?”^52^.

Multimorbidity was determined by a simple count of the following 19 self-reported morbidities: hypertension, cardiovascular disease (atherosclerosis, heart failure, heart attack), stroke, diabetes mellitus, dyslipidemia, anxiety, depression, sleep apnea, arthritis/arthrosis, gastroesophageal reflux, varicose veins of lower limbs, urinary incontinence, liver disease (fatty liver disease, cirrhosis), thyroid disease (hypothyroidism, hyperthyroidism), respiratory disease (asthma, bronchitis), cancer, infertility, polycystic ovary syndrome, and osteoporosis. In this study, we used the definition proposed by The European General Practice Research Network for multimorbidity as any combination of chronic disease with at least one other disease (acute or chronic) or biopsychosocial factor (associated or not) or somatic risk factor^10^, and we also analyzed the prevalence of 3 or more conditions. Complex multimorbidity was defined as the occurrence of three or more chronic conditions affecting three or more body systems or different domains^14^.

The sample size was calculated a posteriori using Epi Info 7 for many explanatory variables, which health self-perception had the highest value (n = 131) for the following parameters: 95% confidence level, 80% power, unexposed: exposed ratio of 33.3: 64.5 being the exposition the fair health self-perception, the percentage outcome in unexposed group 33.3% and risk ratio of 1.81. Therefore, the sample size of the clinical trial (n = 150) was sufficient to cover the purpose of this study.

Descriptive analyses were performed using absolute and relative frequencies, means and standard deviations. The frequency of top ten pairs and triads of morbidities were calculated to describe the most common combinations of conditions. The association between the outcome and independent variables was tested using Pearson’s Chi-squared test and Tendency Chi-squared test. Calculations of relative risks and their 95% confidence intervals (95% CI) were performed using Poisson regression with robust variance. The multiple regression model was adjusted for variables with a *p*-value < 0.20. The significance level was established at 5% (95% confidence level). All analyses were conducted using STATA 12.0^®^. Data entry was performed in duplicate for consistency check using EPI DATA^®^ 3.1.

The study was approved by the Research Ethics Committee of the Clinics Hospital of the Federal University of Goiás (protocol number 747.792). All patients who agreed to participate signed an informed consent.

## Data Availability

We analyzed baseline data of individuals with severe obesity from a randomized clinical trial entitled “Effect of Nutritional Intervention and Olive Oil in Severe Obesity: Randomized Controlled Trial-DieTBra Trial” (registered at ClinicalTrials.gov, NCT02463435). The datasets used and/or analyzed during the current study are available upon formal request to author EA Silveira.
